# The Retrotympanum Revisited By a Volumetric Approach Using Synchrotron-Based X-Ray Phase-Contrast Imaging

**DOI:** 10.1097/MAO.0000000000004884

**Published:** 2026-03-12

**Authors:** Sven Beckmann, Cecilia Lotto, Changling Li, Aleksandra Ivanovic, Raffael Fink, Marco Caversaccio, Anne Bonnin, Lukas Anschuetz

**Affiliations:** a Department of Otorhinolaryngology, Head and Neck Surgery, Inselspital, Bern University Hospital, University of Bern, Bern; b Department of Otorhinolaryngology, Head and Neck Surgery, CHUV, University of Lausanne, Lausanne; c The Sense Innovation and Research Center, Lausanne and Sion, Lausanne; d Paul Scherrer Institut, Swiss Light Source, Villigen PSI; e Hearing Research Laboratory, ARTORG Center, University of Bern, Bern, Switzerland

**Keywords:** Endoscopic ear surgery, Facial recess, Retrotympanum, Sinus tympani, Subtympanic sinus, Suprafacial recess, Synchrotron-based X-ray phase-contrast imaging

## Abstract

**Hypothesis:**

We hypothesize a large anatomic variability with a positive correlation between retrotympanic recess volume and depth classification and an inverse correlation between recess volume and facial nerve distance.

**Background:**

The retrotympanum consists of various bony recesses and is of particular interest given its frequent involvement in cholesteatoma. As the structures are only partly accessible with established imaging techniques due to their size, we propose here to systematically analyze the different retrotympanic subsites and describe topographic relationships using synchrotron-based X-ray phase contrast imaging (SR X-PCI).

**Methods:**

Ten fresh-frozen human temporal bones underwent SR X-PCI at the TOMCAT beamline at the Swiss Light Source. Microtomographic data sets were acquired and segmented for visual inspection. In each specimen, sinus tympani, facial recess, posterior sinus, and lateral tympanic sinus were present and were identified for further analysis.

**Results:**

Among the volumes of retrotympanic recesses, the posterior sinus was on average the smallest and sinus tympani the largest. A positive correlation was observed between the volumes of the sinus tympani and facial recess and their respective A–B–C classifications. Moreover, an inverse correlation between volume and distance to the facial nerve was found for the different retrotympanic recesses. In addition, a previously undescribed recess located above the facial recess, termed the suprafacial recess, was identified in 7 specimens, and its volume was quantified.

**Conclusion:**

SR X-PCI enabled 3D visualization and volumetric analysis of retrotympanic subspaces. Our findings confirmed the complex and variable anatomy of this region and revealed a consistent presence of a previously underreported suprafacial recess.

## Background

The tympanic cavity consists of various bony recesses, with the posterior subspace (retrotympanum) being of particular interest given its frequent involvement in residual cholesteatoma^1^. With the spread of endoscopic ear surgery, the retrotympanum gained special clinical interest, as the different recesses are directly visible with angled endoscopes compared with traditional microscopic approaches^2^. Endoscopic visualization has led to the introduction of a clinical-topographical classification into a superior and inferior part divided by the subiculum and into an anterior and posterior separated part by the facial nerve^3^.

The most well-known recess is the sinus tympani (ST), which was already described by Meckel^4^ in 1820 as a deep recess between the pyramidal eminence, the promontory, and the oval window. The first systematic description was made in 1879 by Steinbrügge^5^ in 37 cases, whereby the ST was present in 35 cases in variable dimensions and was, therefore, considered a normal anatomic variant. Lateral to the facial nerve, in the superior lateral part, lies the facial recess (FR), also described as the suprapyramidal fossa of Sappey and commonly used as a landmark for posterior tympanotomy. In the lateral part below is an additional cavity known as the lateral tympanic sinus (LTS) or Fossula of Grivot^6^.

The first systematic study of the retrotympanum was conducted by Proctor, who designated the different bony bridges in the retrotympanum as ponticulus, subiculum, sustentaculum promontorii, and chordal ridge^7^. In order to standardize the nomenclature in the retrotympanum, renaming the sustentaculum promontorii to finiculus^8^ and the chordal ridge to chordiculus^9^ was recently proposed. In addition, an occasionally well-defined sinus in the area concamerata is recommended to be referred to as the subtympanic sinus nowadays^8^.

Dimensions of retrotympanic subsites have so far been only described by measurements in cadaveric temporal bone dissections or computed tomography (CT) slices in the literature^6,10,11^. As the structures of the retrotympanum are only partly accessible with previous techniques due to their size, we propose here to systematically analyze the retrotympanum with synchrotron-based X-ray phase-contrast imaging (SR X-PCI), allowing detailed submicron structural assessment of both surface and internal tissue architecture. This technique was already used to explore the microstructure of the middle ear ossicles^12^. Herein, we aim to describe the first systematic analysis of the retrotympanum using SR X-PCI and report on volumetric data of the various subspaces and topographical relationships. We hypothesize a large anatomic variability with a positive correlation between recess volume and depth classification and an inverse correlation between recess volume and facial nerve distance.

## Methods

### Samples

This study has been approved by the local ethics committee (Kantonale Ethikkommission Bern, KEK-BE 2016-00887) and the local ethical committee for the Paul Scherrer Institute (Ethikkommission Nordwest-und Zentralschweiz, 2017-00805). We obtained 10 fresh-frozen human temporal bones from anonymous body donors (Institute of Anatomy of the University of Bern) and surgically prepared the specimens for imaging acquisition. All specimens exhibited normal, intact, and variable middle ear anatomy without evidence of middle ear disease. For surgical preparation, the tegmen tympani, the antrum mastoideum, the facial nerve, and the middle ear cavity were skeletonized by removing the mastoid portion. In addition, the soft tissue in the anterior and inferior portions, including the internal carotid artery, jugular bulb, and cartilaginous portion of the Eustachian tube, was removed. Finally, the full peripheral auditory system was left intact, especially the middle ear cavity. This is necessary to minimize the absorption of the X-ray beam by bone during image acquisition.

### Imaging

A multiscale investigation using SR X-PCI was carried out at the TOMCAT beamline (X02DA) at the Swiss Light Source (Paul Scherrer Institute, Switzerland). Due to the sample size, a low-resolution scan overview (effective pixel size of 6.5 μm) was first performed before taking higher-resolution images locally (pixel size of 2.75 μm). The number of needed subscans depended on the size of the sample.

The acquired tomograms were phase retrieved using the Paganin single-distance method^13^ before being reconstructed using the Gridrec algorithm^14^. The parameters used to get the best contrast (ie, the best δ/β ratio) for the acquisitions were δ=1e-7 and β=1e-9 for a propagation distance of 250 mm for the higher resolution scans. After the reconstruction, the subscans were stitched using NRStitcher^15^ to obtain one 3D data set containing the entire peripheral auditory system. Both low-resolution and high-resolution overviews were used in our analysis, depending on the availability of the acquired data.

### Quantitative volumetric analysis

**Figure 1:**
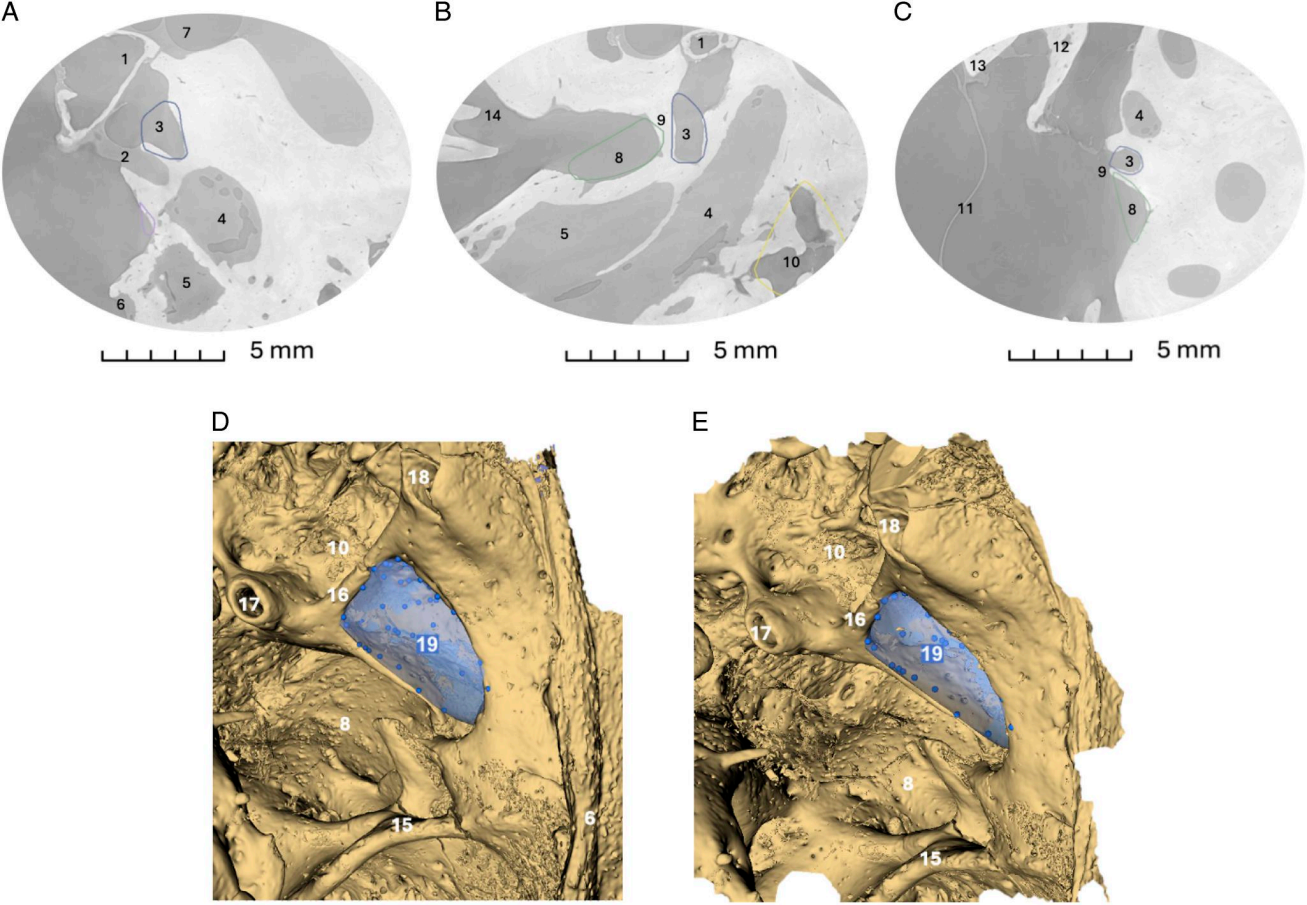
Example of measurements from a single specimen (Specimen 5) of a left ear displayed across all 2D planes. The slices come from 3D grey scale synchrotron-based X-ray phase-contrast microtomographies reconstructed in absorption. (A) Axial view; (B) Sagittal view; (C) Coronal view. 1: Stapes; 2: Tendon of the stapedial muscle; 3: Posterior tympanic sinus; 4: Facial nerve; 5: Stapedial muscle; 6: Posterior anulus; 7: Vestibule; 8: Sinus tympani; 9: Ponticulus; 10: Facial recess; 11: Tympanic membrane; 12: Incus; 13: Malleus; 14: Round window; 15: Subiculum; 16: Chordiculus; 17: Pyramidal process; 18: Chordal eminence; 19: Lateral tympanic sinus.

Both data sets were downsampled using pixel binning to an isotropic voxel size of 11 or 13 μm to reduce computational load. Subsequently, semiautomatic segmentation was performed using the “*Threshold*” tool in the open-source medical image computing platform 3D Slicer to enable 3D visualization^16^. For each specimen, the retrotympanic subspaces were visually identified in the axial, sagittal, and coronal planes of the 2D slices, using the following anatomic landmarks: the facial nerve, chordiculus, chordal eminence, ponticulus, pyramidal eminence, and the posterior annulus of the tympanic membrane (Fig. 1A,B,C). Once a recess was identified, it was annotated in 3D Slicer using the “*Markups*” module by placing a series of points to delineate its boundaries (blue dots in Fig. 1D–E). A 3D volumetric model was then generated with the “*Markups to Model*” tool, allowing volume approximation and subsequent measurement.

The boundaries separating them from the middle ear cavity were defined by placing “*Markup*” points along the bony ridges that limit the recesses. This ensured that the resulting 3D model represented only the recess volume, excluding the volume of the middle ear cavity. For example, in the lateral tympanic sinus shown in Figure 1D–E, “*Markup”* points were placed on the chordiculus medially, on the chordal eminence superiorly, on the bony ridge separating it from the sinus tympani inferiorly, and across the entire surface of the recess. In this way, the generated volume did not include any part of the middle ear cavity.

To assess spatial relationships, the shortest linear distances between each recess and the adjacent segment of the facial nerve were measured using the *“Line”* tool within the *“Markups”* module on the 3D reconstructed images.

### Qualitative analysis

The ST and FR were classified as type A, B, or C according to the system proposed by Alicandri-Ciuffelli et al^9^ and Marchioni et al^17,18^, reported in Table 1 (Supplemental Digital Content 1, http://links.lww.com/MAO/C392), based on their depth in relation to the facial nerve. We then performed the rank correlation analysis (Kendall τ)^19^ between the volume of the ST and FR and their corresponding classification, to confirm the relationship between volumetric extension and depth in proximity to the facial nerve. Subsequently, a second rank correlation analysis was performed between the volumes of all 5 calculated recesses and the minimum linear distance from each to the facial nerve, to assess whether a greater volume is inversely proportional to the distance from the facial nerve. All statistical analyses were performed with R (version 4.5.2, R core team).

## Results

**Figure 2:**
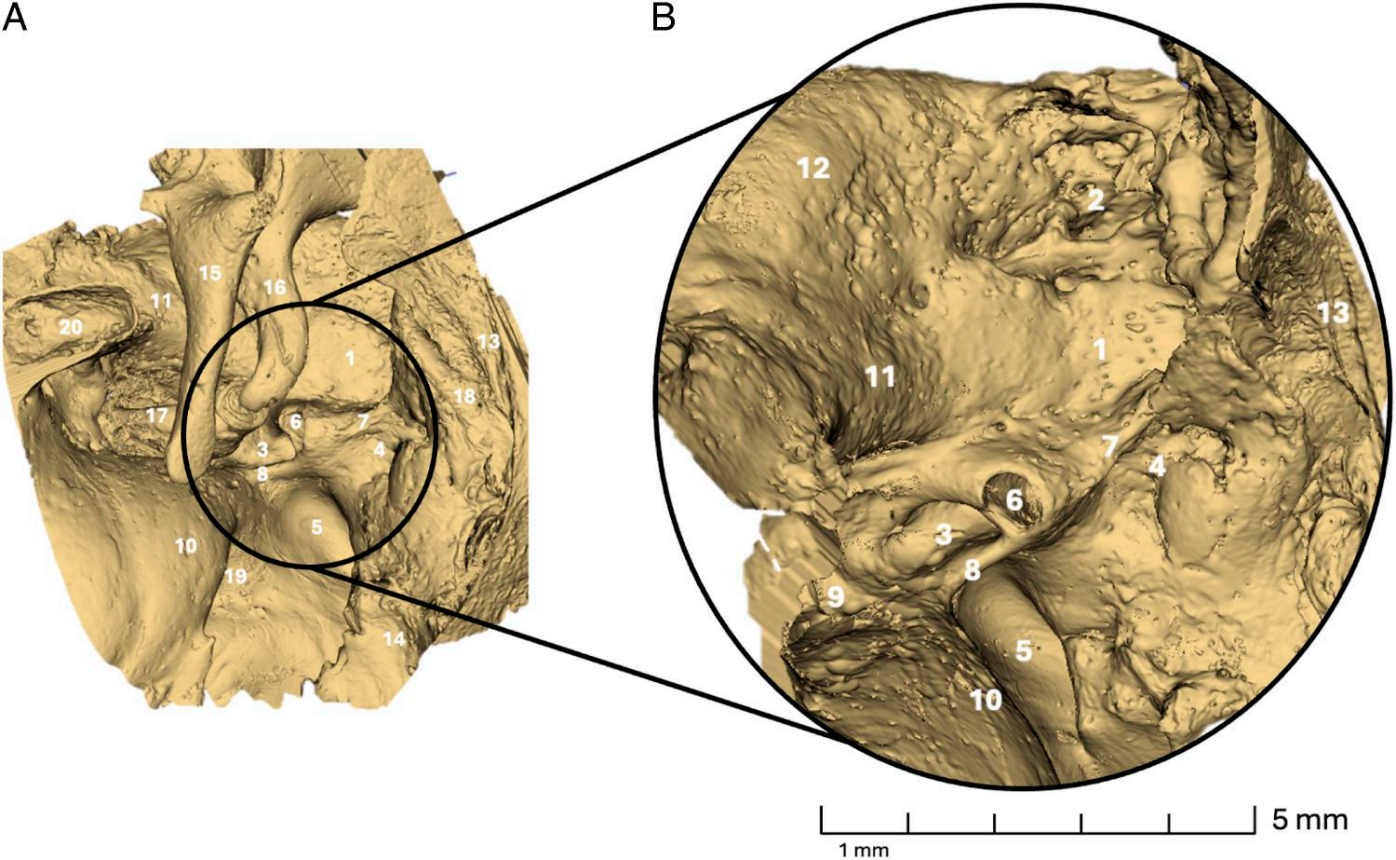
Overview (A) and magnification (B) of a left retrotympanum. 1 FR; 2 Suprafacial recess; 3 PST; 4 LST; 5 ST; 6 PE; 7 Chordiculus; 8 Ponticulus; 9 Footplate; 10 Promontorium; 11 FN; 12 Lateral Canal; 13 Anulus; 14 Area Concamerata; 15 Malleus; 16 Incus; 17 Stapes; 18 Chordal Eminence; 19 RW; 20 Tensor tympani muscle.

**Figure 3:**
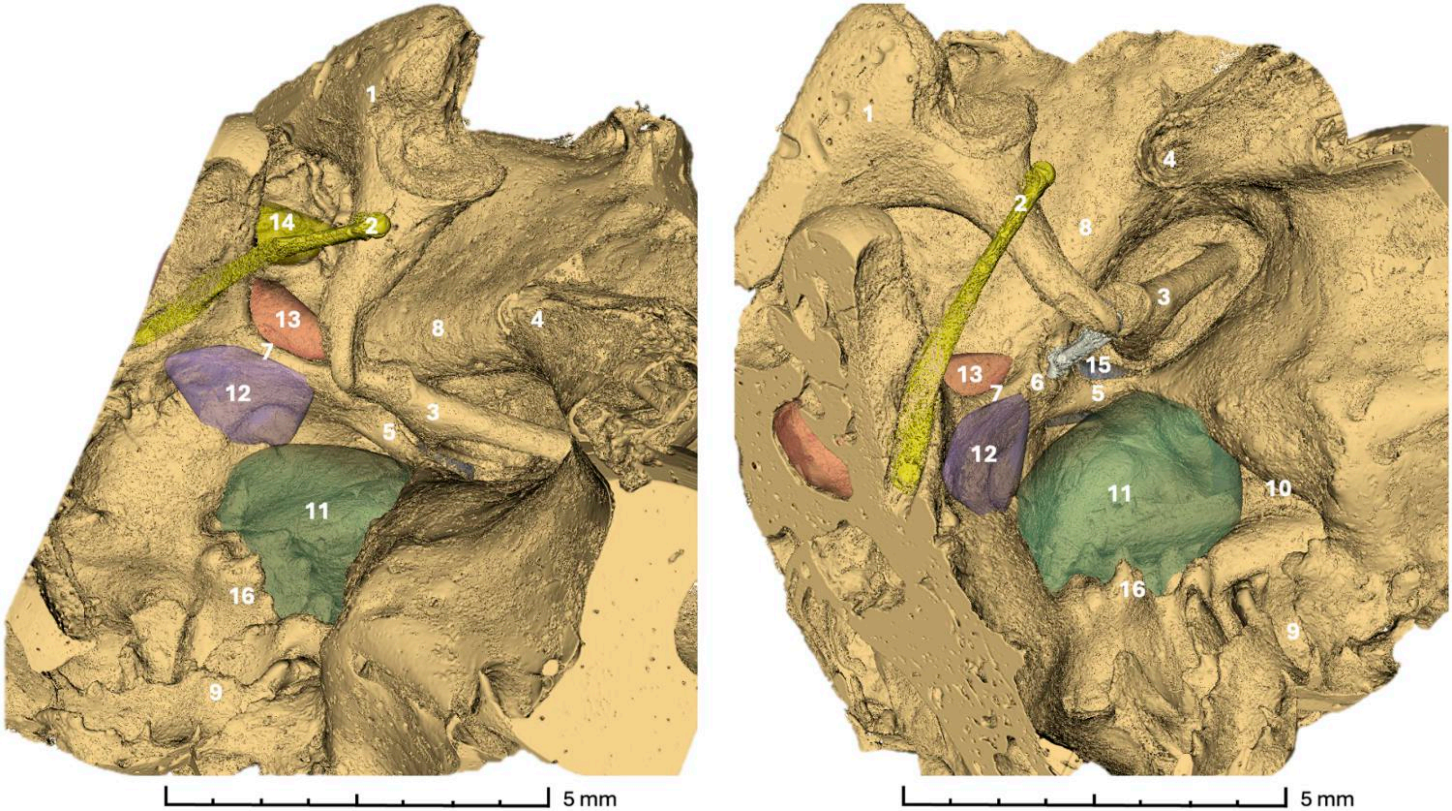
Visualization of a right retrotympanum. 1 Incus; 2 Chorda tympani; 3 Stapes; 4 Cochleariform process; 5 Ponticulus; 6 Pyramidal eminence; 7 Chordiculus; 8 Facial Nerve; 9 Area Concamerata; 10 Round Window; 11 Sinus Tympani; 12 Lateral Sinus Tympani; 13 Facial Recess; 14 Suprafacial Recess; 15 Posterior Tympanic Sinus; 16 Subiculum.

**Figure 4:**
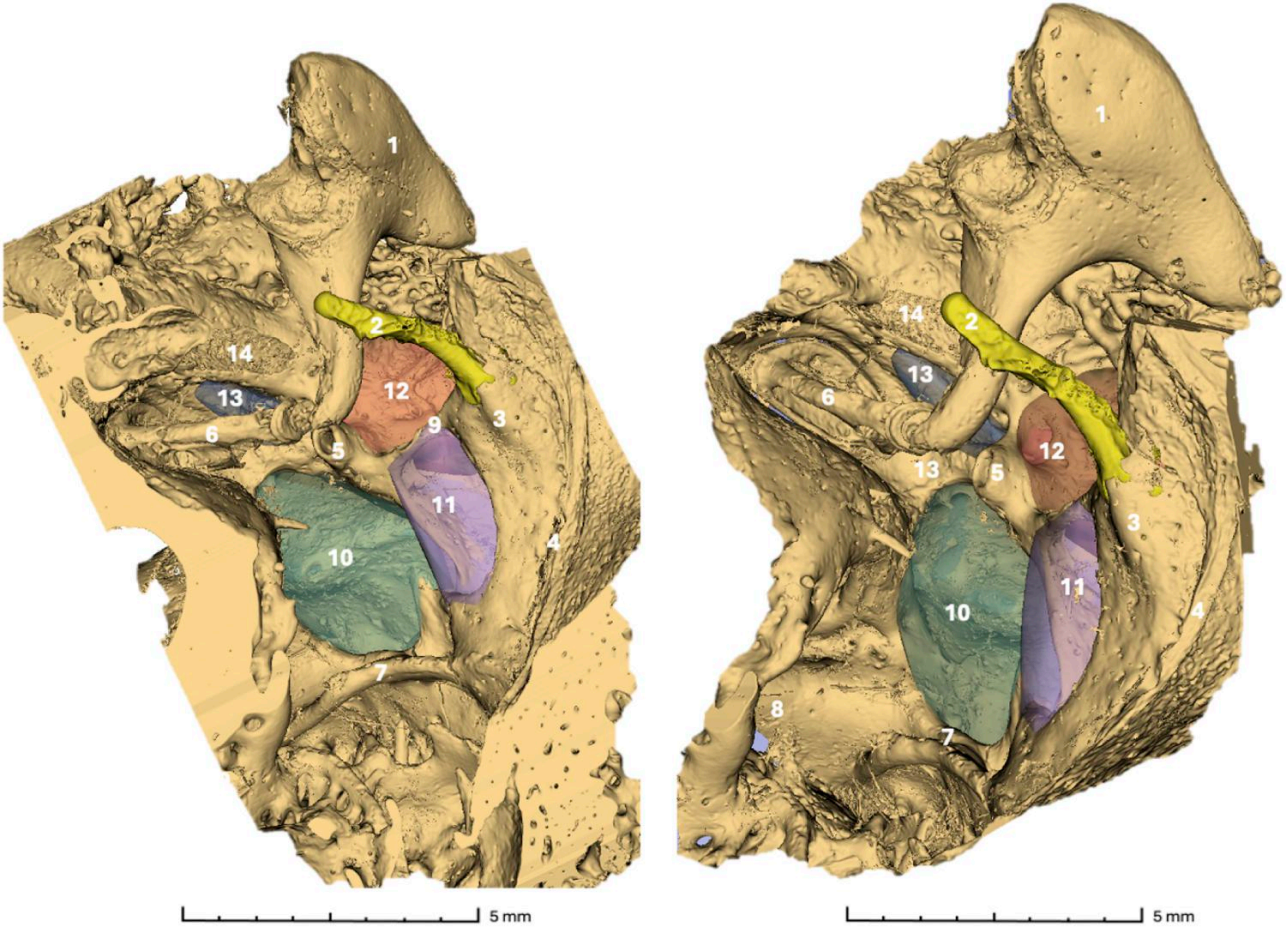
Visualization of a left retrotympanum. 1 Incus; 2 Chorda tympani; 3 Chordal eminence; 4 Anulus; 5 Pyramidal eminence; 6 Stapes; 7 Subiculum; 8 Round window; 9 Chordiculus; 10 ST; 11 LST; 12 FR; 13 PST; 14 FN.

Ten fresh-frozen skeletal human temporal bones were successfully scanned, and tomographic data sets were acquired. The morphology of the temporal bones was segmented semi-automatically and subsequently visually analyzed for each specimen. Landmark structures utilized for orientation included the pyramidal eminence, the osseous canal of the facial nerve, the tendon of the stapedius muscle, the chordal eminence, the chordiculus, the ponticulus, and the subiculum, as seen in Figure 2. In each specimen, the ST, the FR, the posterior tympanic sinus (PST), and the LST were present and selected for volumetric assessment. Figure 3 and Figure 4 provide a 3-dimensional visualization of the retrotympanum, along with the volume highlighted.

**Figure 5:**
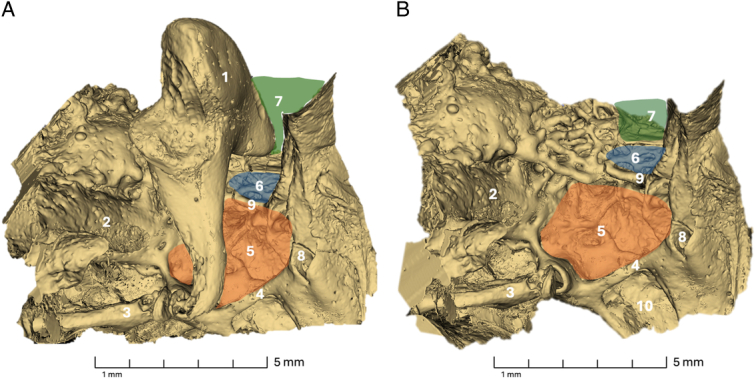
Reconstruction of FR, SFR, and fossa incudis with incus in place (A) and removed (B) (right ear). 1 Incus; 2 FN; 3 Stapes; 4 Chorda tympani; 5 FR; 6 SFR; 7 Fossa incudis; 8 Canaliculus chorda tympani; 9 Osseous spicule separating FR from SFR.

Furthermore, an additional recess was identified and analyzed in 7 specimens. This bony lacuna is located between the FR and the fossa incudis, typically represented by a small cell separating the 2 more prominent structures. It has been designated as the suprafacial recess (SFR), as seen in Figure 5. The SFR should not be confused with the Fossula of Grivot, a distinct anatomic space located laterally and inferiorly to the FR, between the FR and the chordiculus. In contrast, the SFR is positioned superior to the FR, closer to the fossa incudis^20^. This distinction is important for precise anatomic classification and surgical orientation within the retrotympanum.

**Figure 6:**
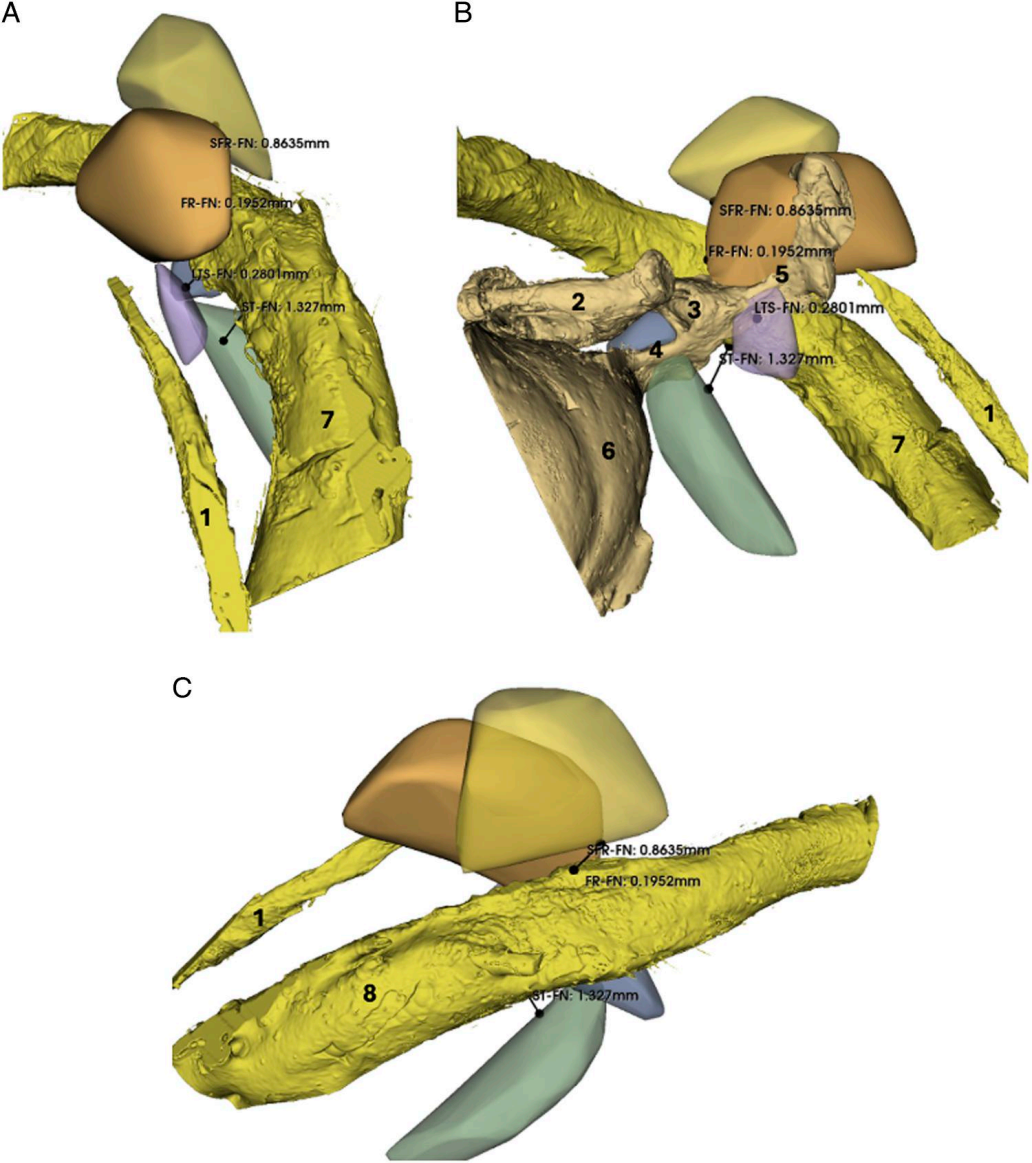
Distances between retrotympanic recesses and FN (left ear). ST (green), PST (blue), LST (violet), FR (orange), and SFR (yellow). (A) lateral-to-medial view; (B) anterior-to-posterior view; (C) posterior-to-anterior view. 1 CT; 2 Stapes; 3 Pyramidal recess; 4 Ponticulus; 5 Chordiculus; 6 Promontory; 7 FN.

Table 2 (Supplemental Digital Content 2, http://links.lww.com/MAO/C393) reports the calculated volumes of the ST, PST, LST, FR, and SFR. The volumes of the ST showed high variability, with a mean of 21.5±16.3 mm³. The mean volume of the PST was 1.4±0.6 mm³. Regarding the lateral retrotympanum, the mean volumes of the FR and LST were 10.8±5.0 mm³ and 4.0±4.6 mm³, respectively. Among these, the PST was the smallest and the ST the largest on average. The ST and FR were then classified as type A, B, or C based on their depth relative to the facial nerve. Among the 10 specimens analyzed, only one ST was classified as type C, whereas none of the FR fell into this category. The most frequently observed type for the FR was type A, found in 7 specimens, while the most common type for the ST was type B, found in 5 specimens. Finally, the minimum linear distances between each recess and the facial nerve were measured (Fig. 6). The mean distance from the facial nerve was 1.2 mm for the ST, 0.6 mm for the PST, 0.3 mm for the FR, and 0.6 mm for the LTS. The SFR was the most distant from the facial nerve, with a mean distance of 1.4 mm.

The correlation analysis between the volume of the FR and its assigned classification (types A, B, or C) revealed a positive correlation (τ=0.62, *P*=0.03), as did the correlation between the volume of the ST and its corresponding classification (τ=0.60, *P*=0.03) (Figure 7, Supplemental Digital Content 3, http://links.lww.com/MAO/C394). Subsequent correlation analyses between the volumes of each recess and their respective minimum linear distances to the facial nerve revealed a consistent inverse relationship, with all correlations showing negative coefficients. Specifically, for the ST, τ=–0.36, *P*>0.1; for the PST, τ=–0.35, *P*>0.1; for the LST, τ=–0.14, *P*>0.5; for the FR, τ=–0.26, *P*>0.1; and for the SFR, τ=–0.33, *P*>0.1. These results indicate a trend whereby larger recess volumes are associated with shorter distances to the facial nerve.

## Discussion

The retrotympanum presents with considerable morphologic variability, as confirmed by our study, which revealed significant differences in the dimensions of the various recesses across all ten specimens. This variability holds important clinical relevance, particularly in middle ear and cholesteatoma surgery, where residual disease frequently involves retrotympanic subsites such as ST and FR^1,21,22^.

Numerous previous studies have aimed to characterize the ST and surrounding structures using diverse methodologies, including cadaveric dissection, histology, and radiologic imaging. Early macroscopic studies by Steinbruegge^5^ and Proctor^7^ laid the anatomic groundwork. Probe-based measurements, such as those by Amjad et al^11^, attempted to quantify depth but were inherently limited by poor visibility and operator variability. More detailed data were obtained from histologic serial sections^23,24^, but tissue processing steps such as fixation and decalcification often introduce artifacts like shrinkage and distortion. Moreover, in histology, distinguishing between adjacent recesses, particularly in the inferior retrotympanum, remains challenging in 2D slices.

CT-based approaches have been used to assess microscopic surgical access routes, especially the retrofacial approach, which is often required to reach deeply located ST recesses^25–27^. Baki et al^28^. and De Abreu and Cruz^29^ highlighted the importance of the lateral bony lip and its removal to improve microscopic visualization, showing that anterior access is feasible only when the ST depth is <1 mm. Despite these advances, conventional CT lacks the spatial resolution to visualize delicate bony partitions or accurately quantify small volumes.

Synchrotron-based X-ray phase-contrast imaging (SR X-PCI) addresses many of these limitations. Unlike conventional imaging or histology, SR X-PCI provides high-resolution, nondestructive, 3D visualization without requiring staining or decalcification. Prior studies have already demonstrated its utility in otologic research; for instance, enabling submicron analysis of ossicular microchannels^12^, bone density pattern^30^, mapping cochlear tonotopy^31^, or assessing aeration pathways^32^. More recently, SR X-PCI has allowed dynamic analysis of ossicular chain movement during auditory stimulation^33^.

Volumetric data on retrotympanic recesses remain limited in the literature. Bekci et al^34^, using high-resolution CT with 1.2 mm slice thickness, reported median ST volumes of 16.1 mm³ (in pneumatized petrous apices) and 8.7 mm³ (in nonpneumatized cases). In our study, SR X-PCI enabled precise volumetric assessment and anatomic characterization of all retrotympanic subspaces (Table 2, Supplemental Digital Content 2, http://links.lww.com/MAO/C393). The volumes measured in our specimens were in most cases slightly higher (mean 21.5 mm³, range 4.3 to 50.2 mm³) than those reported by Bekci, likely due to the superior spatial resolution (∼3 orders of magnitude) of SR X-PCI, which allows detection of small anatomic features that may be missed on conventional CT. Our results further support the findings of Bekci and colleagues, confirming a positive correlation between recess volume and classification depth. Specifically, we observed a positive correlation between the volume and the A to C classification of both the ST and the FR (Figure 7, Supplemental Digital Content 3, http://links.lww.com/MAO/C394), indicating that larger volumes are associated with greater depth and medial extension toward the facial nerve.

In addition, the technique enabled the accurate measurement of the minimum linear distance between each recess and the facial nerve (Fig. 6). For this correlation, a consistent inverse trend was observed: larger recesses were associated with shorter distances to the facial nerve. Notably, this inverse correlation was the largest for the ST. These findings suggest that increased pneumatization may correspond to closer anatomic proximity to critical structures, potentially implying a higher surgical risk.

Similarly, in a recent study by Geneci and Ocak^10^, ST, FR, and the subtympanic sinus were evaluated using micro-CT and classified according to the type A to C classification. After scanning 20 right and 20 left temporal bones, the mean depth reported for the FR was 3.5 mm, 2.7 mm for the ST, and 2.4 mm for the subtympanic sinus, with the highest values observed in type C recesses. Our volumetric findings complement these data and confirm that increased classification depth corresponds with increased volume. Notably, in our cohort, type A was the most common configuration of the facial recess, while type B predominated in the sinus tympani. This distribution differs from that reported by Hool et al^35^, who observed a prevalence of type A across all retrotympanic subtypes in ears affected by cholesteatoma—likely due to impaired middle ear ventilation and reduced pneumatization. In contrast, all ears included in our study were free from middle ear pathology, which may explain the higher anatomic variability and more pneumatized recesses.

In addition to these findings, we identified a distinct recess located above the facial recess and below the fossa incudis in 7 of our specimens. This space likely corresponds to the type II facial recess described by Cheiţă et al^36^, characterized by an accessory cavity superior and lateral to the facial recess. We propose the term “suprafacial recess” for anatomic clarity. Although underreported in the literature, the consistent presence of this recess across specimens suggests it is a regular anatomic variant. Given that cholesteatoma often extends through the posterior mesotympanic route, affecting the ST and FR, the SFR could also be involved in disease spread^37^. While this space appears accessible through a posterior tympanotomy, further studies are needed to evaluate its visibility with a transcanal endoscopic approach.

Limitations of our study must be acknowledged. First, the boundaries between individual recesses are to some extent arbitrary, especially in transitional or confluent spaces. In addition to the fluent boundaries at such high resolutions, the use of 2 data sets for downsampling to a voxel size of 11 or 13 μm harbors the possibility of systematic errors. Second, the number of specimens analyzed is relatively small resulting from the time-consuming process to generate SR X-PCI with limited access to beamtime and synchrotron facilities, limiting statistical power.

## Conclusion

SR X-PCI represents a powerful and innovative tool for the study of retrotympanic anatomy, enabling high-resolution, nondestructive, 3-dimensional visualization and precise volumetric analysis of complex subspaces. This study confirms the highly variable and intricate morphology of the retrotympanum described in previous literature. In particular, a positive correlation between the recess volume and classification depth was demonstrated, as well as an inverse correlation between the recess volume and distance to the facial nerve. Moreover, we constantly identified an SFR in several specimens, which might indicate the presence as a regular anatomic variant. These findings contribute to a better understanding of retrotympanic anatomy and highlight the risk of possible facial nerve involvement in the presence of pathology within deep or large retrotympanic recesses.

## Acknowledgments

The authors acknowledge the Paul Scherrer Institute, Villigen, Switzerland, for providing synchrotron radiation beamtime at the TOMCAT beamline X02DA of the SLS and the Swiss National Science Foundation (SNSF) for funding this research.

The authors wish to thank the Institute of Anatomy, University of Bern, Switzerland, and Mrs. Nane Boemke, especially for the specimens provided.

## Author Contributions

S.B. and C.L.: data collection/management, data analysis, manuscript writing/editing, and final approval of the manuscript; C.L. and R.F.: data collection/management, data analysis, and final approval of the manuscript; A.I.: data collection/management, data analysis, manuscript writing/editing, and final approval of the manuscript; M.C.: protocol/project development, critical review, and final approval of the manuscript; A.B.: protocol/project development, data collection/management, critical review, and final approval of the manuscript; L.A.: protocol/project development, data collection/management, data analysis, manuscript writing/editing, and final approval of the manuscript.

## Supplementary Material

## References

[1] Thomassin JM Korchia D Doris JMD . Endoscopic-guided otosurgery in the prevention of residual cholesteatomas. Laryngoscope 1993;103:939–943. 8361301 10.1288/00005537-199308000-00021

[2] Bowdler DA Walsh RM . Comparison of the otoendoscopic and microscopic anatomy of the middle ear cleft in canal wall-up and canal wall-down temporal bone dissections. Clin Otolaryngol 1995;20:418–422. 8582073 10.1111/j.1365-2273.1995.tb00074.x

[3] Nogueira JF Mattioli F Presutti L Marchioni D . Endoscopic anatomy of the retrotympanum. Otolaryngol Clin North Am 2013;46:179–188. 23566904 10.1016/j.otc.2012.10.003

[4] Meckel JF . Handbuch Der Menschlichen Anatomie Vierter Band. Halle und Berlin: Buchhandlung des hallischen Weisenhauses; 1820.

[5] Steinbruegge H . Ueber Den Sinus Tympani. Zeitschrift für Ohrenheilkunde; 1879.

[6] Parlier-Cuau C Champsaur P Perrin E Rabischong P Lassau JP . High-resolution computed tomographic study of the retrotympanum. Surg Radiol Anat 1998;20:215–220. 9706682 10.1007/BF01628898

[7] Proctor B . LXXXVIII surgical anatomy of the posterior tympanum. Ann Otol Rhinol Laryngol 1969;78:1026–1040. 5350031 10.1177/000348946907800509

[8] Marchioni D Alicandri-Ciufelli M Piccinini A Genovese E Presutti L . Inferior retrotympanum revisited: an endoscopic anatomic study. Laryngoscope 2010;120:1880–1886. 20715093 10.1002/lary.20995

[9] Alicandri-Ciufelli M Fermi M Bonali M . Facial sinus endoscopic evaluation, radiologic assessment, and classification. Laryngoscope 2018;128:2397–2402. 29513386 10.1002/lary.27135

[10] Geneci F Ocak M . Examination of sinus tympani, subtympanic sinus, and facial sinus with MicroCT . Clin Anat 2023;36:414–419. 36268691 10.1002/ca.23968

[11] Amjad AH Starke JJ Scheer AA . Tympanofacial recess in the human ear. Arch Otolaryngol Head Neck Surg 1968;88:131–137. 10.1001/archotol.1968.00770010133003 5662923

[12] Anschuetz L Demattè M Pica A Wimmer W Caversaccio M Bonnin A . Synchrotron radiation imaging revealing the sub-micron structure of the auditory ossicles. Hear Res 2019;383:107806. 31606582 10.1016/j.heares.2019.107806

[13] Paganin D Mayo SC Gureyev TE Miller PR Wilkins SW . Simultaneous phase and amplitude extraction from a single defocused image of a homogeneous object. J Microsc 2002;206:33–40. 12000561 10.1046/j.1365-2818.2002.01010.x

[14] Marone F Stampanoni M . Regridding reconstruction algorithm for real-time tomographic imaging. J Synchrotron Rad 2012;19:1029–1037. 10.1107/S0909049512032864 PMC3480277 23093766

[15] Miettinen A Oikonomidis IV Bonnin A Stampanoni M . NRStitcher: non-rigid stitching of terapixel-scale volumetric images. Murphy R, ed. Bioinformatics 2019;35:5290–5297. 31116382 10.1093/bioinformatics/btz423

[16] Fedorov A Beichel R Kalpathy-Cramer J . 3D Slicer as an image computing platform for the Quantitative Imaging Network. Magn Reson Imaging 2012;30:1323–1341. 22770690 10.1016/j.mri.2012.05.001 PMC3466397

[17] Marchioni D Mattioli F Alicandri-Ciufelli M Presutti L . Transcanal endoscopic approach to the sinus tympani: a clinical report. Otol Neurotol 2009;30:758–765. 19704360 10.1097/MAO.0b013e3181b0503e

[18] Marchioni D Molteni G Presutti L . Endoscopic anatomy of the middle ear. Indian J Otolaryngol Head Neck Surg 2011;63:101–113. 22468244 10.1007/s12070-011-0159-0 PMC3102170

[19] Kendall MG . Rank Correlation Methods, 4th ed. London: Griffin; 1970.

[20] Berrettini S Neri E Ravecca F . Correlations between virtual endoscopy and otoendoscopy of the retrotympanum. Acta Otolaryngol 2002;122:474–478. 12206254 10.1080/00016480260092255

[21] Beckmann S Hool SL Yacoub A . Accuracy of high-resolution computed tomography compared to high-definition ear endoscopy to assess cholesteatoma extension. Otolaryngol Head Neck Surg 2023;169:1276–1281. 37418100 10.1002/ohn.413

[22] Bonali M Fermi M Alicandri-Ciufelli M . Correlation of radiologic versus endoscopic visualization of the middle ear: implications for endoscopic ear surgery. Otol Neurotol 2020;41:E1122–E1127. 32925849 10.1097/MAO.0000000000002787

[23] Saito R Igarashi M Alford BR Guilford FR . Anatomical measurement of the sinus tympani. A study of horizontal serial sections of the human temporal bone. Arch Otolaryngol 1971;94:418–425. 5114950

[24] Ozturan O Miller CC Bauer CA Jenkins HA . Dimensions of the sinus tympani and its surgical access VIA a retrofacial approach. Ann Otol Rhinol Laryngol 1996;105:776–783. 8865772 10.1177/000348949610501004

[25] Pickett BP Cail WS Lambert PR . Sinus tympani: anatomic considerations, computed tomography, and a discussion of the retrofacial approach for removal of disease. Am J Otol 1995;16:741–750. 8572136

[26] Aslan A Guclu G Tekdemir I Elhan A . Anatomic limitations of posterior exposure of the sinus tympani. Otolaryngol Head Neck Surg 2004;131:457–460. 15467617 10.1016/j.otohns.2004.03.028

[27] Chen B Yin S Shen P . The feasibility of the retrofacial approach to the pediatric sinus tympani. Otolaryngol Head Neck Surg 2005;133:780–785. 16274809 10.1016/j.otohns.2005.06.011

[28] Baki FA Dine MBE Saiid IE Bakry M . Sinus tympani endoscopic anatomy. Otolaryngol Head Neck Surg 2002;127:158–162. 12297804 10.1067/mhn.2002.127588

[29] De Abreu CE Cruz OLM . Surgical anatomy of anterior and retrofacial approaches to sinus tympani. Otol Neurotol 2007;28:682–684. 17592400 10.1097/MAO.0b013e318068b298

[30] Ivanovic A Schalbetter F Schmeltz M . Characterizing bone density pattern and porosity in the human ossicular chain using synchrotron microtomography. Sci Rep 2024;14:18498. 39122776 10.1038/s41598-024-69608-9 PMC11315917

[31] Li H Helpard L Ekeroot J . Three-dimensional tonotopic mapping of the human cochlea based on synchrotron radiation phase-contrast imaging. Sci Rep 2021;11:4437. 33627724 10.1038/s41598-021-83225-w PMC7904830

[32] Li H Giese D Rohani SA . Aeration of the Human Prussak’s Space: a 3D synchrotron imaging study. Otol Neurotol 2021;42:E894–E904. 33859141 10.1097/MAO.0000000000003127

[33] Schmeltz M Ivanovic A Schlepütz CM . The human middle ear in motion: 3D visualization and quantification using dynamic synchrotron-based X-ray imaging. Commun Biol 2024;7:157. 38326549 10.1038/s42003-023-05738-6 PMC10850498

[34] Bekci T Hizli O Ozturk M Yildirim G . Quantitative three-dimensional computed tomography analysis of sinus tympani volume in temporal bones with petrous apex pneumatization. Auris Nasus Larynx 2020;47:587–592. 32057525 10.1016/j.anl.2020.01.009

[35] Hool SL Beckmann S Hakim A . Variability of the retrotympanum and its association with mastoid pneumatization in cholesteatoma patients. Eur Arch Otorhinolaryngol 2023;280:131–136. 35695918 10.1007/s00405-022-07465-w PMC9813076

[36] Cheiţă AC Măru N Mogoantă CA Ioniţă E . The recesses of the retro-tympanum. Rom J Morphol Embryol 2010;51:61–68. 20191121

[37] Rosito LS Netto LFS Teixeira AR Da Costa SS . Classification of cholesteatoma according to growth patterns. JAMA Otolaryngol Head Neck Surg 2016;142:168. 26747599 10.1001/jamaoto.2015.3148

