# Supplementary material for: The Retrotympanum Revisited By a Volumetric Approach Using Synchrotron-Based X-Ray Phase-Contrast Imaging
Source: Otol Neurotol. 2026 Mar 12;47(5):e796–803. doi: 10.1097/MAO.0000000000004884 (PMC13155214; doi:10.1097/MAO.0000000000004884)
Supplement: Supplementary file 1 [file mao-47-e796-s001.docx]

|  | ***Type A – Small*** | ***Type B – Deep*** | ***Type C – Very Deep*** |
| --- | --- | --- | --- |
| ***Sinus Tympani*** | No medial or posterior extension relative to the mastoid segment of the facial nerve | Medial extension relative to the mastoid segment of the facial nerve | Posterior extension relative to the mastoid segment of the facial nerve |
| ***Facial Recess*** | Limited to the level of the mastoid segment of the facial nerve | Posterior extension relative to the mastoid segment of the facial nerve | Extending posteriorly and medially to the mastoid segment of the facial nerve |

**Table 1, Supplememtal Digital Content 1.** Radiomorphologic classification of sinus tympani (ST) and facial recess (FR) (adapted from Marchioni^17,18^ and Alicandri-Ciuffelli^9^)
