# Supplementary material for: The Retrotympanum Revisited By a Volumetric Approach Using Synchrotron-Based X-Ray Phase-Contrast Imaging
Source: Otol Neurotol. 2026 Mar 12;47(5):e796–803. doi: 10.1097/MAO.0000000000004884 (PMC13155214; doi:10.1097/MAO.0000000000004884)
Supplement: Supplementary file 2 [file mao-47-e796-s002.docx]

| ***Specimen*** | ***Sinus Tympani*** | ***Type SN*** | ***Posterior tympanic sinus*** | ***Lateral tympanic sinus*** | ***Facial recess*** | ***Type FR*** | ***Suprafacial recess*** | ***FN-ST*** | ***FN-PST*** | ***FN-LST*** | ***FN-FR*** | ***FN-SFR*** |
| --- | --- | --- | --- | --- | --- | --- | --- | --- | --- | --- | --- | --- |
| 1 | 50.2 | C | 1.7 | 11.7 | 18.1 | B | 0.9 | 0.6 | 0.11 | 0.1 | 0.2 | 2.6 |
| 2 | 4.5 | A | 0.9 | 0.8 | 9.3 | A | 6.3 | 1.3 | 0.3 | 0.3 | 0.2 | 0.9 |
| 3 | 14 | B | 1.3 | 1.3 | 16.2 | B | - | 1.6 | 0.4 | 0.4 | 0.2 | - |
| 4 | 22.7 | B | 0.7 | 13.9 | 15.2 | A | 0.8 | 0.9 | 1.1 | 0.5 | 0.2 | 2.2 |
| 5 | 25.3 | B | 1.2 | 1.5 | 5.1 | A | 1.9 | 1.7 | 1.1 | 0.8 | 0.3 | 1.2 |
| 6 | 4.3 | B | 0.5 | 0.7 | 7.5 | A | 1 | 1.9 | 0.7 | 0.2 | 0 | 0.4 |
| 7 | 10.1 | A | 1.9 | 1 | 7.7 | A | 3.1 | 0.8 | 0.9 | 1.7 | 0.6 | 1.4 |
| 8 | 7.4 | A | 1.9 | 4.1 | 2.5 | A | - | 1.3 | 0.2 | 0.3 | 0.4 | - |
| 9 | 26 | B | 2.5 | 1 | 14.8 | B | 0.4 | 1.4 | 0.2 | 1.1 | 0.4 | 1.5 |
| 10 | 50.1 | B | 1.4 | 3.8 | 11.4 | A | - | 0.3 | 0.9 | 0.2 | 0.2 | - |

**Table 2, Supplemental Digital Content 2.** Volumetric measurements different retrofacial recesses (mm³). Depth classification of sinus tympani and facial recess follows the system proposed by Marchioni and Alicandri-Ciuffelli.^8,9,18^ Linear distances (mm) between each recess and facial nerve (FN) are also reported.
