# Supplementary figures and images for: The Retrotympanum Revisited By a Volumetric Approach Using Synchrotron-Based X-Ray Phase-Contrast Imaging
Source: Otol Neurotol. 2026 Mar 12;47(5):e796–803. doi: 10.1097/MAO.0000000000004884 (PMC13155214; doi:10.1097/MAO.0000000000004884)

**A****Facial recess**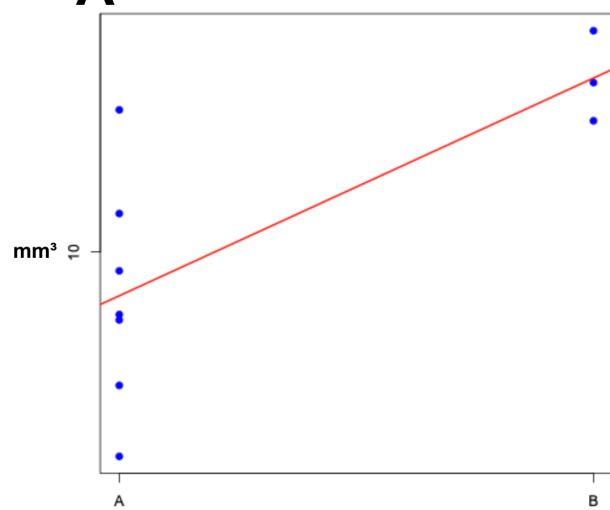**B****Sinus tympani**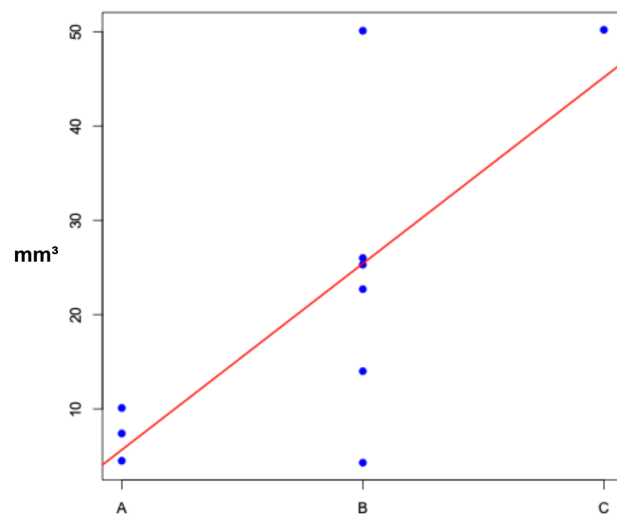

Supplement: Supplementary file 3 [file mao-47-e796-s003.pdf]
